# Anemia in a Cohort of Greek Patients with Sickle Cell Disease: Clinical Insights and Therapeutic Challenges

**DOI:** 10.3390/hematolrep18050075

**Published:** 2026-10-12

**Authors:** Michael D. Diamantidis, Ioanna Argyrakouli, Efthymia Betsi, Alexandra Roupakia, Petroula Krestinidi, Maria Palassopoulou, Omar Zayed, Lazaros Karaloizos, Achilles Manafas

**Affiliations:** Thalassemia and Sickle Cell Disease Unit, Department of Hematology, General Hospital of Larissa, 41221 Larissa, Greece; ioanna-arg@hotmail.com (I.A.); efhmpetsh@gmail.com (E.B.); alroupakia@yahoo.gr (A.R.); p.krestinidi@hotmail.com (P.K.); marpalasso@yahoo.gr (M.P.); omar.zayed32@gmail.com (O.Z.); karaloizoslazaros@gmail.com (L.K.); manafasachilles@gmail.com (A.M.)

**Keywords:** sickle cell disease (SCD), anemia, vaso-occlusive crises (VOC), hydroxyurea (HU), alloimmunization

## Abstract

**Background/Objectives**: Sickle cell disease (SCD) is a multi-systemic hemoglobinopathy affecting many patients worldwide. Clinical manifestations include vaso-occlusive (VOC), aplastic, or hemolytic crises. Anemia of a complex etiology is also very common. However, detailed data regarding the level of anemia in Greek or Mediterranean populations with SCD/β-thalassemia are limited. **Methods**: We retrospectively investigated anemia in 53 SCD patients and described the molecular background regarding mutations in beta and alpha alleles in 49 SCD patients (49/53, representing 92.45% of the cohort). DNA analysis was performed in the Laboratory of Medical Genetics, National and Kapodistrian Athens University. **Results**: The median age was 50 years (range 18–79 years). A total of 20 patients (38%) were diagnosed with severe anemia (Hgb < 8 g/dL), and 6 patients had a very severe degree of anemia (Hgb < 7 g/dL). On the contrary, four patients had very mild or no anemia, depending on their sex. The median hemoglobin levels of the cohort were 8.72 g/dL (range 6.5–13.3 g/dL), while median reticulocyte levels were 183 K/μL (range 18.35–706 K/μL). We found that patients with the beta zero thalassemic allele had statistically decreased Hgb levels (mean Hgb 8.29 g/dL) and thus a more severe degree of anemia compared with SCD patients with a beta plus allele (mean Hgb 9.48 g/dL, *p* = 0.010 < 0.05, Student’s *t* test). **Conclusions**: Our genotype-stratified analysis characterized the molecular basis of anemia in a well-defined cohort of SCD patients. Clinical trials and future research are essential to investigate possible novel drugs for treating anemia and managing its long-term complications in SCD patients.

## 1. Introduction

Sickle cell disease (SCD), a global public health problem according to the World Health Organization (WHO), is the most frequent hemoglobinopathy in the world. The disease affects an estimated 7.74 million people (a 41.40% increase since 2000), with 515,000 affected newborns and a total mortality burden of 376,000 deaths annually [1]. Regarding the SCD burden, 81,100 children under the age of 5 died from SCD-related complications, demonstrating its high morbidity and mortality. Interestingly, the number of actual deaths is roughly 11 times higher than the record of standard single-cause deaths (376,000 total burden vs. 34,400 cause-specific deaths), because complications like organ failure or severe infections are often listed instead of SCD. When analyzing the total mortality impact, SCD ranks as the 12th leading cause of death globally for children under 5 years old [1].

The clinical manifestations of the disease involve most organs of the human body, and despite scientific progress, patients with SCD still die from severe complications. These complications affect major bodily systems, namely hematologic, cerebrovascular, renal, pulmonary, hepatic, skeletal, and cardiovascular [2,3,4,5]. For example, liver complications, cirrhosis, and advanced liver fibrosis are significant causes of morbidity in patients with SCD [6].

The hallmark of the disease is the appearance of red blood cells (RBCs), which transform from flexible, disk-shaped cells into rigid, C-shaped, or “sickle-like” shapes due to the presence of the abnormal hemoglobin S (HbS) [7]. The aforementioned deformity is caused by a single-point missense mutation in the hemoglobin beta (HBB) gene on chromosome 11, defined by the substitution of the amino acid glutamic acid with valine (GAG to GTG) [8]. Hence, sickle cells are elongated, stiff, rigid, and sticky, often obstructing blood flow and being prematurely destroyed, leading to anemia and intense pain (severe crises) [9].

Crises in SCD can either be vaso-occlusive (VOC), aplastic, or hemolytic. Moreover, splenic sequestration is not a rare complication. Hemolysis and episodes of microvascular vaso-occlusion lead to end-organ ischemia–reperfusion injury and infarction [7,10]. Vaso-occlusive events and intravascular hemolysis promote inflammatory processes and redox instability, causing progressive small- and large-vessel vasculopathy [11,12].

The most common type of SCD in Greece is the combination of the SCD allele and the beta thalassemia allele (double heterozygosity), which causes the production of abnormal hemoglobin S. Fewer patients in Greece are homozygous for the SCD allele (homozygous SCD) [13,14]. The most severe form of the disease involves hemoglobin SS [4]. Moreover, hemoglobin SC includes the second most common type of SCD and occurs when a patient inherits one sickle cell gene (“S”) and one abnormal gene called “C”, producing another type of abnormal protein. Patients with HbSC are usually located in Sub-Saharan Africa, Mediterranean countries, the Middle East, and parts of India and often have less severe anemia because their blood cell counts are higher. Nevertheless, they can still experience painful crises [15].

Rarer types of SCD occur with the inherited combinations of one sickle cell gene (“S”), along with genes for other rare abnormal hemoglobins, such as “D”, “E”, and “O”. HbSD [16,17] and HbSO [16] usually result in symptoms similar to sickle cell anemia, causing moderate to severe anemia and occasional pain episodes. Furthermore, patients with HbSE [18] are usually located in Southeast Asia, Turkey, and Saudi Arabia. They have mild to moderate anemia and an increased risk of infections [18].

The two most common types of SCD in Greece (homozygous SCD and SCD allele/beta thalassemia allele) have almost the same clinical manifestations; these patients are thus treated with the same therapeutic protocols, aiming to prevent SCD crises and related acute and chronic complications of all organs [4,7]. Recently, there has been emerging data regarding advanced liver fibrosis [6], hepatic clinical manifestations [19], and renal dysfunction in patients with SCD [20], proving the poly-systematic nature of the disease. Interestingly, sepsis due to infections, liver failure, and stroke were the three predominant causes of death in a large cohort of Greek patients with SCD [3].

We retrospectively identified the degree of anemia in a cohort of Greek patients diagnosed with SCD because detailed data on the level of anemia in Greek or Mediterranean populations with SCD/β-thalassemia are either totally lacking or very limited. We also evaluated the molecular determinants associated with anemia in these patients, mainly through a genotype-stratified analysis. The objective of our study was to link the molecular background of SCD with the level of anemia.

## 2. Materials and Methods

A retrospective observational analysis regarding the levels of anemia of 53 patients with SCD was conducted (median value and range) based on the patients’ medical records. All laboratory examinations of patients at our hospital (General Hospital of Larissa, Greece) are stored electronically, so comparisons can be made for the same patient over time. Most patients had many measurements of hemoglobin, hematocrit, and reticulocytes for all of the studied years; thus, the median value was calculated, and the range was provided. The hemoglobin measurements were obtained during steady-state disease, meaning that the patients were not under any type of SCD crisis.

The median duration of the follow-up, which included full clinical and hematological evaluations, was 20 years for every patient, with at least two evaluations conducted yearly (every six months), including for the 5 patients who died. Many patients were followed up even more closely, with evaluations conducted every three months for more than 20 years. For the few patients who received transfusions, data collection was always performed before the transfusions, so these data reflect pre-transfusion values.

Regarding the hemoglobin range, we preferred to use the threshold of 1 g/dL to differentiate each subgroup of patients based on the level of anemia. This is because an increase of 1 g/dL is a major criterion of response and a primary endpoint in many similar studies, like the BEYOND study involving non-transfusion-dependent thalassemic patients, though this comparison is indirect and involves a different hemoglobinopathy [21,22].

The eligibility criteria included SCD diagnosis, either in a homozygous (S/S) or b-thalassemia allele/S allele state. Most patients (49/53) had a confirmed diagnosis based on molecular DNA analysis. The few patients without a DNA analysis were diagnosed based on their electrophoresis and clinical manifestations (crises). All patients were frequently followed up over several years, as stated above.

DNA analysis was performed in the Laboratory of Medical Genetics, National and Kapodistrian Athens University, using standard methods such as Amplification-Refractory Mutation System Polymerase Chain Reaction (ARMS-PCR), Polyacrylamide Gel Electrophoresis (PAGE), Gap Polymerase Chain Reaction (GAPPCR), Restriction Enzyme Polymerase Chain Reaction (RE-PCR), and Sanger sequencing. The DNA analysis results are kept in separate files in our department, making them easy to access.

Statistical analysis was conducted using SPSS 21.0. The Shapiro–Wilk normality test was applied because genotypic data was only available for 49 patients (less than 50). The Shapiro–Wilk test is the preferred statistical test for checking normality when the sample size is less than 50 cases (*n* < 50) [23]. Moreover, Student’s *t*-test was used for comparisons, with *p* < 0.05 set as the level of statistical significance. We also performed a subgroup analysis regarding the possible link between the patients’ genotype and degree of anemia.

The thalassemia allele IVSI-110, frequently found in Greek patients, is generally considered a weak beta plus gene, though its clinical behavior is closer to that of a beta zero variant. Therefore, we performed two types of analysis: in the first, IVSI-110 was considered a beta plus allele, and in the second, a beta zero allele. Due to severe anemia, the only three patients homozygous for SCD were classified as beta zero (a thorough analysis is presented in the results).

This study was conducted in accordance with the Declaration of Helsinki. All data used in the study are available from our department (DNA results are presented with patients’ initials) upon request. No identifiable information is provided for any of the patients (full names) due to personal data restrictions. All patients provided consent before DNA analysis was performed. The study was approved by the Scientific and Ethics Committee of the General Hospital of Larissa, Greece.

## 3. Results

A total of 53 patients with SCD were included in the study, with 25 males and 28 females. The median age was 50 years (range 18–79 years). A total of 48 patients are still alive today, whereas 5 of them have died. The first patient died from cerebral hemorrhage (55 years), the second from septicemia (73 years), the third from intrahepatic cholangiocarcinoma (55 years, incidental death, not SCD-related), and the remaining two from acute crisis due to SCD (33 and 40 years, respectively). Only 4 patients in the cohort did not receive hydroxyurea (HU), while the remaining 49 received HU on a regular basis (15–20 mg/kg of body weight). Most patients (50/53) had a combination of the SCD allele and the beta thalassemia allele (SCD/beta thalassemia or HbS/beta thalassemia), and only three were homozygous for SCD. Genetic analysis was available for 49 patients (46 with SCD/beta thalassemia and 3 with homozygous SCD). The four remaining patients were diagnosed based on their electrophoresis and clinical manifestations (crises), all of whom had both alleles (SCD/beta thalassemia).

The range of anemia according to the seven levels of hemoglobin is depicted in Table 1, while Table 2 shows the median values and the patients’ ranges of hemoglobin and reticulocytes, along with the genotype.

Six patients had a very severe degree of anemia (Hgb < 7 g/dL). A significant percentage of the cohort (20/53, 38%) showed a severe level of anemia (Hgb < 8 g/dL); six of these patients had Hgb < 7 g/dL. On the contrary, four patients had either very mild or no anemia (Hgb > 11.10 g/dL) depending on their sex (Hgb NR: females, 12–16 g/dL; males, 13.50–17 g/dL) (Table 1). The three patients homozygous for HbS/HbS had severe anemia (median Hgb: 7.10, 7.40 and 7.76) even though they did not carry a beta-thalassemic allele. They were thus included in the “beta zero” arm for the genotype–anemia comparison due to meeting the clinical criterion of severe anemia.

We also observed that another 38% (20/53) of our cohort suffered from a moderate level of anemia (Hgb between 8.10 and 10 g/dL), whereas nine SCD patients (9/53, 17%) had a mild level of anemia (Hgb 10.10–11 g/dL) (Table 1).

The median Hgb level of the whole cohort was 8.72 g/dL [mean: 8.90 g/dL ± 1.60 (SD)] with a range of 6.50 g/dL (minimum) to 13.30 g/dL (maximum). Moreover, the median reticulocyte level of the whole cohort was 183 K/μL [mean: 229.41 K/μL ± 141.41 (SD)] with a range of 18.35 K/μL (minimum) to 706 K/μL (maximum). The normal range of reticulocytes was 25 K/μL–80 K/μL.

Most patients had an elevated median number of reticulocytes due to chronic hemolysis and the compensatory mechanisms of the bone marrow (BM) to overcome the anemia. However, four patients had reticulocyte levels lower than 80 K/μL, representing a normal number of reticulocytes or hypoplastic marrow, and thus did not have viral or microbial infection. In the latter cases, the compensatory mechanisms of the BM to overcome anemia were not effective.

The 23 patients with the beta zero thalassemic allele had statistically decreased hemoglobin levels [mean Hgb levels of 8.29 g/dL ± 1.27 (SD)] and thus a more severe degree of anemia compared with the 26 patients with a beta plus allele [mean Hgb levels of 9.48 g/dL ± 1.75 (SD); *p* = 0.010 < 0.05, Student’s *t* test]. Student’s *t* test was applied (normal distribution, Shapiro–Wilk, *p* = 0.060 and 0.859, both >0.05).

The same rationale was observed when the IVSI-110 was considered a beta zero rather than a beta plus allele. In the latter case, the 39 patients with the beta zero thalassemic allele had statistically decreased hemoglobin levels [mean Hgb levels of 8.62 g/dL ± 1.44 (SD)] and thus a more severe degree of anemia compared with the 10 patients with a beta plus allele [mean Hgb levels of 10.08 g/dL ± 1.92 (SD); *p* = 0.011 < 0.05, Student’s *t* test]. Student’s *t* test was applied (normal distribution, Shapiro–Wilk, *p* = 0.092 and 0.937, both >0.05).

The lower median Hgb thresholds for the beta zero allele were 8.29 g/dL and 8.62 g/dL statistically in both comparisons. The three patients homozygous for HbS/HbS had lower Hgb levels than the aforementioned thresholds (median Hgb: 7.10, 7.40, and 7.76, all lower than 8.29 g/dL and 8.62 g/dL), thus clinically justifying their inclusion in the “beta zero” arm for the genotype–anemia comparison.

Only three patients in our cohort received chronic red blood cell (RBC) transfusions. The first patient (HbS/IVSI-110) had an enlarged spleen with a size of around 21 cm, and RBC transfusions were performed to decrease it. Patients with SCD often have functional asplenia due to splenic infarcts. Nevertheless, in fewer cases, the thalassemic element prevails for unknown reasons and causes splenomegaly. The two other patients were older, and RBC transfusions were used to treat their worsening anemia.

Interestingly, in the last 5 years, no novel allo-antibodies have been identified in our unit, mainly due to our department’s policy requiring all RBC transfusions to be matched by phenotype.

## 4. Discussion

The degree of anemia in patients with SCD can vary from mild to severe (Hgb < 8 g/dL) or very severe (Hgb < 7 g/dL) for many reasons, as seen in our studied cohort. The nature of anemia in SCD is multifactorial. Chronic hemolysis due to hemoglobinopathy is one factor contributing to anemia in such patients [4]. The destruction of sickle cells leads to various levels of hemolytic anemia among SCD patients [24]. An increased number of reticulocytes is produced by the BM as a compensatory mechanism to treat anemia [25]. Thus, patients with SCD usually have an elevated number of reticulocytes, as seen in most of our cohort. In addition, the hemoglobin measurements in the present study were obtained during a steady state of the disease. Acute complications of SCD, like vaso-occlusive crises, infections, and aplastic crises, along with hospitalization when necessary, might substantially influence the severity of anemia [4].

The genetic combination of the SCD and beta thalassemia alleles is very common in Greece. Patients with complete lack of beta chains (beta zero, β0) have more severe anemia compared to SCD patients, who have beta plus (β+, or β++) alleles. Examples of beta zero alleles are CD39, IVSI-n1, and IVSII-1 G>A, whereas typical beta plus alleles include IVSI-6 T>C and IVSII-745 C>G [14]. The most common allele in Greece, IVSI-110, is typically classified as beta plus. However, it is clinically a weak beta plus allele, resembling a beta zero allele, and causes a severe level of anemia [3,14].

Based on the findings of our study, we correlated molecular data (genotype) with the degree of anemia and showed that patients with a beta zero allele statistically had a more severe degree of anemia compared with patients with a beta plus variant (median Hgb level of 8.29 g/dL vs. 9.48 g/dL). Thus, a stratified genotypic analysis affecting the level of anemia in our patient cohort yielded our main finding: a beta zero hemoglobin variant is statistically correlated and associated with lower hemoglobin levels and worsening of anemia. However, the genotype analysis was not conducted in four patients.

According to the literature, either bacterial or viral [26] infections and sepsis are also important factors contributing to anemia in SCD patients [27]. Viral infections associated with parvovirus B19 cause particularly severe anemia with high morbidity in SCD patients, along with aplastic crises in the BM, often requiring RBC transfusions [27].

The basic therapeutic approach for SCD in 2026, which also reflects our center’s policy, is to avoid regular transfusion and to instead prescribe HU to prevent crises, though there are exceptions [4,28]. Few patients with SCD receive regular transfusions, especially those over the age of 70 (more than 6 RBC units yearly). RBC transfusions are also applied in cases of acute chest syndrome or other acute complications associated with severe anemia and VOCs [10,24,25,28].

SCD alloimmunization nevertheless remains a serious complication of transfusions that can lead to life-threatening acute and delayed reactions, along with severe hemolytic anemia, especially in SCD patients, who receive transfusions less frequently (less than six RBC units yearly) [29,30,31]. Higher alloimmunization rates are observed in SCD patients due to RBC antigenic differences between donors and recipients. However, it remains unclear why few SCD patients develop alloantibodies compared to the whole population [29,30]. To prevent alloimmunization, our center requires extended antigen-matched donor RBC typing for all SCD patients. Nevertheless, it has been reported that very few SCD patients continue to develop alloantibodies due to a high degree of polymorphisms in immunogenic antigens in individuals of African ancestry worldwide [29,30,31]. No alloantibodies have been detected in the SCD patients of our department in the last 5 years due to the improved transfusion policy, as highlighted above.

HU is the cornerstone in treating SCD patients. Even though the drug prevents SCD crises by increasing the levels of HbF in the BM, it also causes dose-dependent marrow suppression, which is generally manageable and reversible. Severe myelosuppression is the most frequently reported adverse reaction, but it is not observed to the same extent in all patients. Thus, there is sometimes a significant reduction in hemoglobin, white blood cells, and platelets in SCD patients who receive HU [2,32]. Drug response levels and clinical manifestations also vary among SCD patients [4,10]. Most patients in the present study (49/53, 92%) receive HU on a chronic basis (15–20 mg/kg of body weight), and only four did not receive HU; however, these patients exhibit various degrees of anemia, ranging from severe to less severe. The latter shows that HU is not the only factor influencing anemia in such patients. There is a large unmet need for novel disease-modifying treatment approaches, either alone or in combination with HU, not only to prevent SCD complications but also to increase hemoglobin levels, which is crucial in such patients [32,33].

Most patients with SCD have functional asplenia due to SCD infarcts in the spleen [10,24,25]. On the contrary, the thalassemic element prevails in a few patients with SCD/thalassemia for unknown reasons. These patients might have splenomegaly or even hypersplenism, which causes severe anemia.

Beyond our findings, we discuss the current drug landscape within the literature. Unfortunately, drugs that initially received approval from the European Medicines Agency (EMA) were withdrawn for several reasons. This caused anxiety, frustration, and disappointment for patients and the clinicians treating them. Voxelotor (an inhibitor of HbS polymerization, HOPE clinical trial, NCT03036813) was approved by the Food and Drug Administration (FDA) in 2019 [34] but was withdrawn by the company globally in September 2024 and by the FDA/EMA after a high number of deaths occurred at malaria-endemic sites [35]. Before its withdrawal, voxelotor was on the market for 4 months in Europe.

The P-Selectin inhibitor crizanlizumab (monoclonal antibody, SUSTAIN clinical trial, NCT01895361) received initial approval both by the FDA and EMA [36], but it was suspended only in Europe by the EMA in August 2023 [37] due to failure to reach clinically meaningful efficacy endpoints. However, it has been reported that crizanlizumab helps prevent VOCs in SCD patients [38,39] and treats priapism [40,41,42], which is a serious complication of SCD. The drug is now only on the market in the United States.

Further research involving drugs like etavopivat and NDec is being conducted. The positive results from the Phase 3 portion of the Phase 2/3 HIBISCUS clinical trial (NCT04624659) for the drug etavopivat are encouraging. Etavopivat reduced the incidence of VOCs, with the median time to first VOC almost doubling (38.4 weeks vs. 20.9 weeks for placebo); it also improved hemoglobin levels, with 48.7% of patients receiving 400 mg of etavopivat achieving an Hgb increase of >1 g/dL at week 24 compared to 7.2% for the placebo (NCT04624659). The analysis of the results is ongoing.

Another promising epigenetic drug for SCD is NDec [a combination of Nd-decitabine and tetrahydrouridine (THU)]. The drug functions primarily as a potent inducer of fetal hemoglobin (HbF) through non-cytotoxic inhibition of DNA methylation. Its mechanism of action directly addresses the root cause of SCD—the polymerization of hemoglobin S (HbS)—by reactivating the expression of the gamma-globin gene, which has been silenced after infancy in SCD patients (NCT05405114, ASCENT1 clinical trial). The results of the study are under analysis. There is a great unmet need for well-designed clinical trials focusing on drugs that can increase Hgb in SCD patients.

There is growing interest in machine-learning methods for predicting anemia severity and classifying genotype–phenotype relationships in hemoglobinopathies. Thus, predictive models—such as machine-learning classifiers incorporating genotype, reticulocyte counts, and longitudinal hemoglobin trajectories—may support prospective risk stratification for anemia severity in patients with SCD. Such approaches could be explored in future multicenter studies involving larger and more diverse cohorts. Clinical implementation of these models would require independent validation, attention to data quality and interpretability, and appropriate consideration of patient safety, privacy, and legal accountability [31,32,33].

An important limitation of our study is its retrospective nature. Prospective studies focus on the present in relation to the future and are preferred over retrospective studies for solid scientific conclusions. Another limitation of our study is that, besides HU, the patients with SCD received more drugs due to having concomitant diseases. These other drugs could have affected the level of anemia in our patient cohort, though this was difficult to assess and was not investigated. A detailed analysis of the role of concomitant medications in morbidity and mortality in patients with hemoglobinopathies has been published previously in a national well-defined cohort [3].

Other limitations of our work include the small sample size, the single-center design, the lack of evaluation of the levels of HbF and/or renal dysfunction, the absence of the study of infections and/or inflammatory markers, and the lack of thorough investigation of splenectomy and of the incidence of alloimmunization. Few patients receive chronic transfusions to improve their anemia, usually after the age of 65 or 70. Elevated HbF levels in SCD patients usually increase hematocrit levels by inhibiting abnormal HbS from clumping together under low-oxygen conditions, although this increase is not always clinically meaningful for many reasons. Chronic renal dysfunction in SCD also contributes to anemia, and elderly patients usually show more severe degrees of anemia, frequently requiring transfusions. There are no conclusive data regarding the impact of sex on the levels of anemia in SCD patients.

Regarding the small sample size, a power analysis calculator indicated an ideal sample size of 210 patients (105 beta zero and 105 beta plus). The statistical criteria for statistical significance were met in our study (*p* < 0.05), even with 53 patients. SPSS was used due to its ability to detect a statistical difference even with smaller sample sizes. We also applied the universally accepted methodology in statistics. Moreover, even though HU, splenectomy, and the transfusion burden were either analyzed in the results or commented on in the discussion, a more detailed analysis would better explain their role in anemia in SCD. Finally, all of these aforementioned factors could be confounding variables influencing the level of anemia in our patients. Nevertheless, we feel that this could be an objective of a future study.

## 5. Conclusions

In conclusion, we highlighted that SCD patients with a beta plus allele statistically had a less severe degree of anemia compared with patients with a beta zero variant. Our retrospective observational study underlines an association between the beta zero genotype and lower hemoglobin levels in our cohort of SCD patients. A significant percentage of our SCD patients had a severe level of anemia (Hgb < 8 or even <7 g/dL). Since anemia contributes to long-term SCD complications, novel drugs for treating anemia in SCD patients are essential.

Patients with SCD eagerly anticipate novel drugs that can simultaneously prevent VOCs and increase Hgb levels.

## Figures and Tables

**Table 1:** The range of anemia in patients according to 7 levels of hemoglobin and the corresponding patient numbers for each category.

Hemoglobin Range (g/dL)	Number of Patients with SCD and Corresponding Percentage
6–7	6 (11.32%)
7.10–8	14 (26.42%)
8.10–9	10 (18.87%)
9.10–10	10 (18.87%)
10.10–11	9 (16.98%)
11.10–12	1 (1.89%)
>12	3 (5.66%)
**Total**	**53 (100%)**

**Table 2:** Median values and range of hemoglobin and reticulocytes.

Patient Number Sex—Year of Birth	Median Hemoglobin (g/dL)	Hemoglobin Range (g/dL)	Median Reticulocyte Range (K/μL) NR 25–80 K/μL	Genotypes for Beta and Alpha Alleles
1 F—1984	10.50	10.20–11	363 (308–375)	HbS/IVSI-110/-a^3.7^
2 M—1967	10.70	10.20–11	370 (308–388)	HbS/IVSI-110
3 F—1958	9.40	8–11.30	unavailable	unavailable
4 F—1960	9	8.10–9.70	279 (233–353)	HbS/IVSII-1 G>A
5 M—1998	8.35	6.50–9.70	138 (9–290)	HbS/IVSI-110
6 M—1947	9.40	7.90–12	179 (164–204)	unavailable
7 F—1967	8.50	7.80–8.90	112 (98–158)	HbS/IVSI-n1
8 F—1976	8.80	6.60–9.70	46 (29–82)	HbS/IVSI-110
9 M—1987	8.60	7.20–10.30	75 (34–118)	unavailable
10 F—1967	7	6.60–7.30	303 (169–317)	HbS/IVSI-n1
11 M—1965	8	7.40–8.10	unavailable	HbS/CD39
12 F—1976	6.60	6.20–7.60	313 (294–332)	HbS/IVSI-110
13 F—1976	7.80	6.30–8.20	392 (392–392)	HbS/IVSI-110
14 F—1983	6.60	5.70–7.60	266 (125–486)	HbS/IVSII-745 C>G
15 M—1960	10.40	8.40–12.40	103 (81–119)	HbS/CD5 del CT/-a^3.7^
16 F—1982	13.30	12.40–13.60	117 (111–122)	HbS/IVSI-6 T>C
17 F—1956	9.20	6.90–10.70	122 (92–160)	HbS/δβ Turkish
18 M—1979	9.20	7.50–10.80	302 (209–404)	HbS/IVSI-110
19 M—1984	7.10	6.50–8.10	116 (43–333)	HbS/CD39/-a^3.7^
20 M—1990	11.30	10.80–12.30	149 (138–154)	HbS/IVSI-110
21 F—1979	9.90	6.40–11.30	377.5 (155–471)	HbS/CD39
22 M—1982	9.95	8.20–11	233 (129–391)	HbS/δβ Lepore
23 M—1986	10.10	7.60–11.20	205.5 (104–360)	HbS/δβ Lepore
24 F—1978	7	5.40–10.10	227 (42–927)	HbS/IVSI-n1
25 F—1978	8.05	7.10–8.70	265 (130–374)	HbS/CD39
26 M—1966	11	10.20–11.40	132 (132–132)	HbS/CD8-AA/-a^3.7^
27 F—1974	8.15	6.60–9.10	91 (5–287)	HbS/IVSI-110
28 F—1976	7.90	6.70–9.30	72.5 (38–181)	HbS/IVSII-745 C>G
29 M—2008	11	7.70–12	157 (113–190)	HbS/IVSI-5 G>A
30 M—1966	7.20	6.40–7.40	18.35 (18.35–18.35)	unavailable
31 M—1982	10.20	9.70–11	157 (40–238)	HbS/IVSII-1 G>A
32 M—1979	7.10	6.60–7.90	249 (130–340)	HbS/HbS
33 F—1952	7.90	5.60–9	183 (176–263)	HbS/CD39/-a^3.7^
34 M—1999	7.70	4.60–10.10	212 (87–298)	HbS/IVSI-n1
35 F—1965	7.50	5.60–9.10	258.5 (214–310)	HbS/364G>C (HBD LOS ANGELES)
36 M—1974	7.20	4.60–9.40	310 (80–749)	HbS/CD39
37 M—1969	9	8.10–9.70	151 (108–240)	HbS/IVSI-110
38 F—1994	7.40	6.70–12.40	523 (84–934)	HbS/HbS/ααα
39 M—1978	12.10	9–12.80	128 (68–196)	HbS/IVSI-6 T>C
40 M—1953	10.65	7.80–12.50	101 (77–130)	HbS/IVSI-6 T>C
41 F—1981	8.70	7.70–9.50	706 (597–961)	HbS/CD39
42 F—1951	7.73	6–10	125 (91–195)	HbS/IVSI-110
43 F—1950	7.76	5.30–9.20	176 (37–332)	HbS/HbS
44 M—1979	9.90	8–11.10	585 (468–631)	HbS/CD39
45 F—1974	6.71	5.70–8.10	150.5 (109–208)	HbS/IVSI-110/ααα
46 F—1985	10.50	8.40–11.10	452 (271–584)	HbS/IVSI-110
47 F—1984	8.72	6–9.80	470 (249–582)	HbS/IVSI-110
48 M—1985	9.75	8.70–10.30	314.5 (208–332)	HbS/IVSI-5 G>A
49 M—1972	12.05	8.67–13.10	243.5 (176–331)	HbS/IVSI-110/poly A
50 F—1976	9.56	8.74–11.44	174 (125–223)	HbS/IVSI-110
51 F—1981	9.45	8.50–10.10	174 (154–244)	HbS/IVSII-745 C>G/poly A
52 F—1984	7.62	6–8.50	233 (71–350)	HbS/CD39
53 M—1976	6.50	6.10–7.20	100 (80–125)	HbS/44bp del

## Data Availability

All data in the study are available from our department (DNA results are presented with the patients’ initials) upon request. The laboratory results of each patient, labelled with their initials, are also available in separate Excel files. No identifiable information is provided for any of the patients (full names) due to personal data restrictions. This is also described in the Section 2 of the manuscript.
